# Receipt of medications for opioid use disorder among youth engaged in primary care: data from 6 health systems

**DOI:** 10.1186/s13722-021-00249-3

**Published:** 2021-07-07

**Authors:** Sarah M. Bagley, Laura Chavez, Jordan M. Braciszewski, Mary Akolsile, Denise M. Boudreau, Gwen Lapham, Cynthia I. Campbell, Gavin Bart, Bobbi Jo H. Yarborough, Jeffrey H. Samet, Andrew J. Saxon, Rebecca C. Rossom, Ingrid A. Binswanger, Mark T. Murphy, Joseph E. Glass, Katharine A. Bradley, PROUD Collaborative, José Szapocznik, Jane M. Liebschutz, Brian K. Ahmedani, Bethann Mangel Pflugeisen, Robert P. Schwartz, Angela L. Stotts, Thomas F. Northrup, Viviana E. Horigian, Angela J. Silva

**Affiliations:** 1 Boston Medical Center, Boston University, Boston, USA; 2 Nationwide Children’s Hospital, Abigail Wexner Research Institute, Columbus, USA; 3 Center for Health Policy and Health Services Research, Henry Ford Health System, Detroit, USA; 4 Department of Psychiatry, Henry Ford Health System, Detroit, USA; 5 Kaiser Permanente Washington Health Research Institute, Seattle, USA; 6 Division of Research, Kaiser Permanente Northern California, Oakland, USA; 7 University of Minnesota Medical School/Hennepin Healthcare, Minneapolis, USA; 8 Kaiser Permanente Northwest Center for Health Research, Portland, USA; 9 Center of Excellence in Substance Addiction Treatment and Education, VA Puget Sound Health Care System/University of Washington School of Medicine, Seattle, USA; 10 Health Partners Institute/University of Minnesota, Minneapolis, USA; 11 Colorado Permanente Medical Group, Kaiser Permanente Colorado, The University of Colorado School of Medicine and The Kaiser Permanente Bernard J. Tyson School of Medicine, Aurora, USA; 12 MultiCare Institute for Research and Innovation, MultiCare Health System WA, Tacoma, USA

**Keywords:** Opioid use disorder, Adolescents, Young adults, Medication for opioid use disorder, Buprenorphine, Naltrexone

## Abstract

**Purpose:**

Little is known about prevalence and treatment of OUD among youth engaged in primary care (PC). Medications are the recommended treatment of opioid use disorder (OUD) for adolescents and young adults (youth). This study describes the prevalence of OUD, the prevalence of medication treatment for OUD, and patient characteristics associated with OUD treatment among youth engaged in PC.

**Methods:**

This cross-sectional study includes youth aged 16–25 years engaged in PC. Eligible patients had ≥ 1 PC visit during fiscal years (FY) 2014–2016 in one of 6 health systems across 6 states. Data from electronic health records and insurance claims were used to identify OUD diagnoses, office-based OUD medication treatment, and patient demographic and clinical characteristics in the FY of the first PC visit during the study period. Descriptive analyses were conducted in all youth, and stratified by age (16–17, 18–21, 22–25 years).

**Results:**

Among 303,262 eligible youth, 2131 (0.7%) had a documented OUD diagnosis. The prevalence of OUD increased by ascending age groups. About half of youth with OUD had documented depression or anxiety and one third had co-occurring substance use disorders. Receipt of medication for OUD was lowest among youth 16–17 years old (14%) and highest among those aged 22–25 (39%).

**Conclusions:**

In this study of youth engaged in 6 health systems across 6 states, there was low receipt of medication treatment, and high prevalence of other substance use disorders and mental health disorders. These findings indicate an urgent need to increase medication treatment for OUD and to integrate treatment for other substance use and mental health disorders.

**Supplementary Information:**

The online version contains supplementary material available at 10.1186/s13722-021-00249-3.

## Introduction

Opioid-related deaths continue to increase in the United States among adolescents and young adults (youth) [1]. Between 1999 and 2016, the opioid-related mortality rate increased 252% among 15–19 year olds [2]. Similar to the adult population, the rise in deaths is driven by illicitly-manufactured fentanyl, which is about 100 times more potent than morphine and 50 times more potent than heroin [2, 3]. Emergency department visits and intensive care unit admissions related to opioid poisonings among youth have also increased [2].

Rising opioid-related deaths have led to efforts to increase access to medication for opioid use disorder (OUD) for all ages [4, 5]. Medication is considered the gold standard for treatment of OUD and improves OUD patient outcomes, including abstinence, retention in care, and survival [6–9]. Although federal regulations limit access to methadone as a treatment for OUD for youth younger than 18 years, two of the FDA-approved medications for OUD (i.e., buprenorphine and naltrexone) can be prescribed in primary carethat care for youth (PC; e.g., office-based settings).

The American Academy of Pediatrics and the American Society for Addiction Medicine recommend that youth with OUD be treated medication [6, 10, 11]. Despite these recommendations, youth with OUD are often not treated with medication. In studies of both publicly and commercially insured youth with a diagnosis of OUD, only 4.5% of adolescents younger than 18 years and one in four young adults 18–22 years old received medications for OUD within three months of diagnosis [12, 13]. Recent national U.S. data indicate that population-based rates of buprenorphine prescriptions decreased among 15–34 year olds from 2009 to 2018, in contrast to all other age groups [14]. None of these studies examined whether patients were engaged in PC. Engagement in PC may represent an important opportunity to offer medications for OUD to youth; most youth in the US see a PC provider and some have long-standing trusting relationships with them.

In the present study, we evaluated the prevalence of OUD and office-based medication treatment for OUD in a large sample of youth and young adults aged 16–25 (hereafter “youth”) who received PC in one of 6 health systems across 6 states. The objectives of this study were to describe, among youth engaged in PC, (1) the prevalence of documented OUD, (2) the prevalence of office-based medication treatment for OUD, and (3) patient characteristics associated with OUD medication treatment.

## Methods

### Design and sample

This was a cross-sectional, three-year, study using secondary data from Phase 1 of the PRimary care Opioid Use Disorders (PROUD) trial. The PROUD trial was a pragmatic, cluster-randomized implementation trial testing whether a collaborative care model for office-based addiction treatment increased use of medication for OUD in PC (2014–2016) [15]. PROUD Phase 1 was a preliminary study to identify potential health systems to participate in the trial and assess the feasibility of cohort identification and data collection [16, 17]. Six of eleven health systems participating in Phase 1 provided data for the present study of youth: Kaiser Permanente (KP) Washington, KP Northwest, KP Northern California, KP Colorado, Health Partners, and MultiCare. These included 5 integrated health delivery and insurance systems with access to claims data for care received *outside* the system and one fee-for-service community health system serving a mixed urban, suburban, and rural population (MultiCare). Sites represented 6 states (Minnesota, Wisconsin, Colorado, California, Oregon, and Washington). Data on patient demographics and clinical characteristics were obtained from electronic health records (EHR) and insurance claims. Medication treatment was based on pharmacy dispensings in the EHR and claims data (5 integrated health systems) or medication orders in the EHR (1 health system)—referred to as prescriptions hereafter, as well as procedure codes (all sites). Data were not available for methadone treatment in Outpatient Treatment Programs (OTPs).

Patients were eligible if they had at least 1 PC visit in fiscal years (FY) of 2014–2016, and were 16–25 years old at the time of their first PC visit. Data were ascertained from electronic health records (EHR) and insurance claims data for the FY of their first PC visit during the study period (“index FY” hereafter). Youth were included in analyses for their index FY. Youth were divided into 3 age groups: 16–17 years, 18–21 years, and 22–25 years. These groups were selected to allow comparisons across developmental stages based on prior work that has shown differences [18].

### Primary outcomes

The primary outcomes were (1) a diagnosis of OUD and (2) one or more prescription(s) for office-based OUD medication treatment (hereafter “treatment”) documented in the index FY. OUD was defined using International Classification of Disease-9th edition (ICD-9-CM; until September 30, 2015) or ICD-10-CM diagnostic codes (starting October 1, 2015). Codes for both “active” and “in remission” OUD were included as providers can differ in their use of the codes (Additional file 1: Table S1). A prescription for OUD medication was defined as documentation of 1 or more dispensings (5-sites), orders (1-site), or procedure codes (all sites) for buprenorphine approved for OUD (transmucosal or implanted) or naltrexone (oral or injectable) in the index FY. Secondarily, we report on 2 or more prescriptions and/or procedures in a FY as a proxy for medication taking.

### Patient characteristics

Patient demographics included gender, age, race/ethnicity, and insurance type at the time of first PC visit. Clinical characteristics were collected in the index FY and included ICD9/10 diagnoses of other substance use disorders (tobacco, alcohol, cannabis, and stimulants), opioid overdose, and psychiatric diagnoses (depression, anxiety, attention deficit disorder, serious mental illness, and eating disorder).

### Analysis

We first characterized the demographic and clinical characteristics of youth with and without documented OUD in the index FY. Among youth with documented OUD, we described characteristics across the three age groups (16–17, 18–21, and 22–25 years). The prevalence (95% confidence intervals [CIs]) of prescriptions for each type of OUD medication (buprenorphine, injectable naltrexone, and oral naltrexone), as well as no OUD treatment, were described graphically across age groups. Finally, we compare the prevalence (95% CIs) of demographic and clinical characteristics in patients with OUD who received medication treatment and those who did not, in the overall sample.

The study was approved by the Kaiser Permanente Washington Institutional Review Board (IRB), and all sites ceded to the Kaiser Permanente Washington IRB except MultiCare who used MultiCare’s IRB for review and approval.

## Results

### Characteristics of youth with documented opioid use disorder

Among 303,262 PC patients 16–25 years old, 2,131 individuals had a documented OUD diagnosis in the index FY. The prevalence of documented OUD was higher with increasing age from 0.16% among 16–17 year olds, to 0.67% among 18–21 year olds, and 1.02% among 22–25 year olds.

Youth with OUD were predominantly male and White, with a substantial burden of tobacco, alcohol, cannabis, and stimulant use disorders (Table 1). Over 3% had a documented opioid overdose in the FY. Table 2 compares demographic and clinical characteristics in youth with OUD across the 3 age groups. Rates of documented substance use and mental health comorbidity tended to be higher in younger patients (Table 2).

**Table 1:** Characteristics of youth age 16–25 years with and without opioid use disorder (OUD)

	OUD (N = 2131)	No OUD (N = 301,131)
	%	%
Gender		
Female	40.7	56.1
Race/ethnicity		
Hispanic	8.7	15.0
White	77.0	57.1
Black/African American	3.1	7.8
Asian	1.7	10.2
Native American/Alaska Native	0.7	0.4
Hawaiian/Pacific Islander	0.4	0.7
Multiracial	4.7	3.1
Other	0.9	1.0
Unknown	2.8	4.8
Insurance type^a^		
Medicare	0.1	0.4
Commercial	82.7	83.1
Medicaid and other state subsidized	12.3	10.2
Uninsured	4.8	6.3
Tobacco use disorder	53.5	5.3
Alcohol use disorder	24.6	1.4
Cannabis use disorder	32.9	1.6
Stimulant use disorder	27.8	0.3
Opioid overdose	3.6	0.0
Depressive disorders	43.4	12.2
Anxiety disorder	47.4	13.7
Serious mental illness^b^	10.1	1.2
Attention Deficit Disorder	13.1	6.0
Eating disorder	1.9	0.6

^a^Excludes one health system with missing insurance data

^b^Serious mental illness defined as schizophrenia and bipoloar disorder

**Table 2:** Characteristics of youth 16–25 years with opioid use disorder stratified by age

	16–17 years (N = 119) %	18–21 years (N = 664) %	22–25 years (N = 1348) %
Gender			
Female	55.5	41.9	38.8
Race/ethnicity			
Hispanic	17.7	9.2	7.7
Caucasian	68.1	75.8	78.4
Black/African American	2.5	2.9	3.3
Asian	2.5	2.1	1.5
Native American/Alaska Native	0.0	0.9	0.6
Hawaiian/Pacific Islander	0.0	0.2	0.5
Multiracial	9.2	5.1	4.0
Other	0.0	0.9	1.0
Unknown	0.0	3.0	3.0
Insurance type^a^			
Medicare	0.0	0.0	0.2
Commercial	83.0	86.9	80.6
State subsidized	14.8	10.5	13.0
Uninsured	2.3	2.5	6.2
Tobacco use disorder	42.9	56.3	53.0
Alcohol use disorder	45.4	24.0	23.1
Cannabis use disorder	64.7	40.5	26.3
Stimulant use disorder	31.9	30.3	26.3
Opioid overdose	2.5	3.5	3.7
Depressive disorders	77.3	43.4	40.4
Anxiety disorder	65.6	46.2	46.3
Serious mental illness^b^	15.1	9.3	10.1
Attention Deficit Disorder	31.9	13.0	11.6
Eating disorder	3.4	3.2	1.2

^a^Excludes one health system with missing insurance data

^b^Serious mental illness defined as schizophrenia and bipoloar disorder

### Proportion of those with documented OUD with medication treatment

**Fig. 1:**
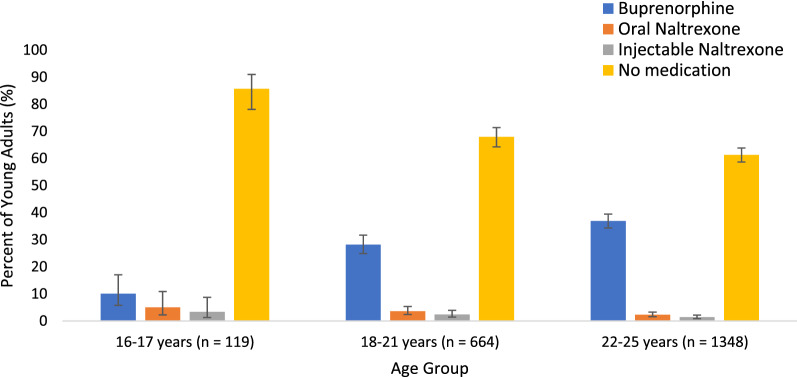
Proportion of youth with opioid use disorder who receive medication treatment with buprenorphine or naltrexone

Overall, 35% (95% CI 33–37%) of youth with OUD received OUD treatment at some time during the index FY. Among 16–17 year-olds with OUD, 14% received treatment: 10% buprenorphine, 5% oral naltrexone, and 3% injectable naltrexone (some received more than one medication) (Fig. 1). For 18–21 year-olds, 32% received treatment: 28% buprenorphine, 4% oral naltrexone, and 2% injectable naltrexone. For 22–25 year-olds, 39% received treatment: 37% buprenorphine, 2% oral naltrexone, and 1% injectable naltrexone.

Overall, 86% of youth receiving buprenorphine had at least two prescriptions for buprenorphine. In contrast, only 46% of youth receiving injectable naltrexone and 33% of youth receiving oral naltrexone had at least two prescriptions for naltrexone.

### Youth with documented OUD: comparison of those with and without medication treatment

In the overall sample with OUD, youth who had OUD medication treatment were less likely than those without medication treatment to have documented depressive disorder (36% vs. 48%), anxiety disorder (42% vs. 50%), serious mental illness (6.9% vs. 12%) or an eating disorder (0.8% vs. 2.5%) (Table 3). There were no differences in the prevalence of sex, race/ethnicity, insurance status, overdose or tobacco or other substance use disorder in those with and without medication treatment. The age stratified results can be found in Additional file 2: Appendix S1.

**Table 3:** Prevalence of demographic and clinical characteristics and 95% confidence intervals in youth with opioid use disorder (OUD) with and without documented treatment with medications for OUD (N = 2131)

	Medication treatment^a^ (n = 752) %, (95% CI)	No treatment (n = 1379) %, (95% CI)
Female	38 (35, 42)	42 (39, 45)
Race/ethnicity		
Hispanic	8.8 (6.9, 11)	8.7 (7.3, 10)
Caucasian	79 (76, 82)	76 (74, 78)
Black/African American	1.9 (1.0, 3.1)	3.8 (2.9, 5)
Asian	1.2 (0.5, 2.3)	2.0 (1.4, 2.9)
NA/AN	0.5 (0.1, 1.4)	0.7 (0.3, 1.3)
Hawaiian/Pacific Islander	0.8 (0.3, 1.7)	0.1 (0.02, 0.5)
Multiracial	3.9 (2.6, 5.5)	5.1 (4, 6.4)
Other	0.8 (0.3, 1.7)	0.9 (0.5, 1.6)
Unknown	3.1 (1.9, 4.6)	2.7 (1.9, 3.7)
Insurance^b^		
Medicare	11 (8.7, 13)	13 (11, 16)
Commercial	85 (83, 88)	81 (78, 83)
State subsidized	0 (0, 0.5)	0.2 (0.02, 0.7)
Uninsured	3.8 (2.5, 5.4)	5.6 (4.3, 7.2)
Tobacco UD	56 (52, 59)	52 (50, 55)
Alcohol UD	21 (18, 24)	26 (24, 29)
Cannabis UD	34 (31, 38)	32 (30, 35)
Stimulant UD	30 (26, 33)	27 (25, 29)
OD	3.3 (2.2, 4.9)	3.7 (2.8, 4.8)
Depression	**36 (32, 39)**	**48 (45, 50)**
Anxiety	**42 (38, 45)**	**50 (48, 53)**
Serious mental illness^c^	**6.9 (5.2, 9.0)**	**12 (10, 14)**
ADD	11 (9.1, 14)	14 (12, 16)
Eating disorder	**0.8 (0.3, 1.7)**	**2.5 (1.8, 3.5)**

*NA/AN* Native American/Alaskan American, *OD* opioid overdose, *UD* use disorders, *ADD* attention deficit disorder

Bolded results are significant at p < 0.05

^a^One or more prescriptions or procedure codes for buprenorphine and/or naltrexone in the fiscal year of index visit

^b^Excludes one health system with missing insurance data

^c^ Serious mental illness defined as schizophrenia and bipoloar disorder

## Discussion

Documented diagnosis of OUD increased with age in this observational study of youth 16 to 25 years old engaged in PC in 6 large U.S. health systems. Among youth with documented OUD, approximately 2 in 3 had depressive or anxiety disorders, half had a tobacco use disorder, 2 in 5 had cannabis use disorders, and 1 in 3 had alcohol or stimulant disorders documented in their EHRs or health insurance claims. The prevalence of office-based OUD medication treatment among youth with OUD increased with age: fewer than 1 in 6 of youth ages 16–17 received buprenorphine or naltrexone while approximately 1 in 3 of those 18–25 years received medication treatment. Buprenorphine was more commonly prescribed compared to naltrexone among all age groups and was more commonly refilled or reordered after the initial prescription. Youth with OUD who were treated with buprenorphine or naltrexone were less likely than those who were not to have mental health disorders documented in EHR or claims data in the same FY.

The prevalence of medication treatment of OUD was higher in our overall sample than in previous studies of youth in large commercial and public insurance cohorts between 2001 and 2018 [12, 13, 19]. In those studies, 4.7% of youth under 18 years covered by Medicaid and 9.7% of youth 16–17 year with commercial insurance received OUD medications in the three and six months after diagnosis of OUD, respectively. In our sample, about 14% of 16–17 year-olds had at least one prescription for OUD medication in a 1 year period. Similarly, young adults in our sample (18–25 years) had a higher prevalence of OUD treatment than in prior studies. Hadland et al. found that 22–31% of young adults with Medicaid and commercial insurance received medications for OUD within 3 months of an OUD diagnosis [12]. In our sample, 32–39% of young adults received medications for OUD in a 12 month period. One potential explanation for the higher prevalence of OUD treatment in our study than in prior studies of youth and young adults, is that youth in our study might have been more engaged in medical care. Our study sample included only youth who had a PC visit in the same year. Further, OUD diagnoses could have occurred within addiction treatment programs in most study health systems. Prior studies of youth with nonfatal overdose have found a low prevalence of medication treatment of OUD [18, 20, 21]. In our study, youth with and without treatment of OUD had comparable rates of nonfatal OD. Of note, these other studies evaluated receipt of medication after a new OUD diagnosis or nonfatal OD. In this study, we report the prevalence of OUD medications in the same FY as the OUD diagnosis or nonfatal OD.

In this study, 86% of young people who started buprenorphine received more than one prescription while only half who started injectable naltrexone received more than one prescription. While injectable naltrexone is an effective medication for treating OUD, patients must be abstinent from opioids prior to starting which is a challenge for many patients [22]. Although these barriers in naltrexone initiation have been well described in adult populations [23–25] there are fewer data about naltrexone treatment in youth [12]. Youth in this study were treated with oral naltrexone as often as injectable naltrexone*.* Those prescriptions could represent a trial before a planned transition to injectable naltrexone, but our data do anot allow further clarification of this. FDA’s approval of buprenorphine starting at age 16, but approval of naltrexone starting at age 18, may also impact decisions about medication choices. There is a need for a more nuanced understanding of how patients, providers, and families are making decisions about starting and continuing OUD medication treatment.

The prevalence of co-occurring other substance use disorder and mental health disorders was very high in this sample of youth with OUD, particularly for 16–17 year olds. Fifty-four percent of youth with OUD also had a tobacco use disorder. A recent study finding that only ~ 5% of youth with nicotine use disorder received pharmacotherapy and/or counseling. Ref. [26] highlights the importance of treating all substance use disorders in youth once they are engaged in treatment for OUD. More than half of the youth with OUD in our study had documented anxiety or depressive symptoms, higher than a prior study of youth in an outpatient substance use treatment program where the prevalence was less than 25% [27]. The higher prevalence of co-occurring disorders in the present sample may reflect the study sample. Some of the study health systems had internal treatment for mental health and substance use disorders, which could have led to increased documentation and treatment. It is possible that some patients could have been diagnosed with OUD in the process of mental health or substance use disorder treatment. However, this high prevalence also highlights the urgency of providing treatment for mental health and SUD in addition to OUD. The prevalence of mental health conditions was lower in patients treated with medications for OUD. This may indicate that mental health providers—including prescribers such as psychiatrists—are often not trained to treat youth with OUD with medications. Given the high prevalence of mental health disorders, further work is needed to ensure that providers treating mental health conditions in youth are prepared to offer evidence-based treatment.

In this study, more than half of the 16–17 year olds with an OUD were female. However, the proportion of youth with OUD who were female decreased as age increased. Although this is not consistent with one study of Medicaid-insured youth with OUD [12], other studies of youth experiencing nonfatal opioid overdose have found a higher proportion of females in younger age groups, with decreases in the proportion who are female as age increases [18, 21]. These sex- and age-based differences in youth with OUD may be the result of secular trends and warrant future study to ensure that age-appropriate, tailored interventions are offered to females and males.

Despite the higher treatment prevalence in this sample compared to prior studies, there remains a significant opportunity to improve access to and engagement in medication treatment for youth with OUD and co-occurring mental health disorders. Offering OUD treatment in PC is not only safe and effective [28–30], but is also likely more convenient and less stigmatizing for patients. Moreover, PC and specialty physicians can treat the patient’s OUD in the context of their other health needs. Future work should focus on identifying the provider characteristics associated with diagnosis and treatment of OUD; such information could inform improvements in systems of care for this population. For those youth receiving specialty mental health services or other SUD treatment, OUD treatment can be integrated into their other mental health and SUD care. Most SUD, including OUD, typically first manifest in the age group that is the focus of this work. Prioritizing early treatment of OUD would likely move these young people into a recovery trajectory sooner in their lifespan to prevent a lifetime of disability.

### Limitations

There are limitations to this study. First, it is cross-sectional, and we were not able to identify incident OUD diagnoses or study the temporality of any associations. In addition, we could not determine whether OUD treatment was prescribed from PC or specialty care settings. The optimal approach for identifying outpatient prescriptions in EHR-based health services research is to use pharmacy medication dispensing data; one health system in this study only had medication orders from the EHR. Despite also using procedure codes to capture medications administrated in the office at all sites, either data source may have resulted in some misclassification of treatment status. We were not able to identify methadone treatment of OUD. Thus, it is possible that we may have underestimated OUD treatment to some extent, particularly for those 18 years and over. In addition, the sample of 16–17 years olds was limited. We previously reported OUD treatment variation by site, and believe much of the observed variation is true differences in treatment practices at the sites [16]. The high prevalence of alcohol use disorder (AUD) in this sample raises the possibility that some participants were prescribed naltrexone for AUD and not OUD. Although over 80% of patients received 2 or more buprenorphine prescriptions—suggesting that buprenorphine was at least initiated by the patient—it is possible that for some patients the two prescriptions represented the induction prescription and the first home prescription. This would lead to an overestimate of how many patients engaged in OUD medication treatment. Five of the health systems in this analysis have integrated mental health care, so that these youth could have greater access to mental health and substance use care than other youth. This may have led to increased opportunity for and documentation of substance use and mental health diagnoses. As a result, our findings may not generalize to other health care settings. Finally, health systems participating in this study were exploring participating in a future implementation trial treating OUD in PC. Thus, they may have been more open than other health systems to providing medications for OUD.

## Conclusions

In this study of over 300,000 patients 16–25 years old from 6 health systems, 0.7% had a documented OUD. Among patients with OUD, 65% had no documented OUD treatment. Significant opportunity exists to improve access to OUD treatment among affected youth. Moreover, the high prevalence of other substance use and mental health disorders among youth with OUD, indicate an urgent need for treatment of OUDs that also addresses polysubstance use and mental health disorders.

## Supplementary Information

**Additional file 1: Table S1. **International Classification of Diseases, Ninth and Tenth Revision, Clinical Modification codes used to define Opioid Use Disorder (OUD).

**Additional file 2: Appendix S1.** Prevalence of demographic and clinical characteristics and 95% confidence intervals in youth with opioid use disorder (OUD) with and without documented treatment with medications for OUD stratified by age (N=2131).

## Data Availability

The current data use agreements we have in place with the sites for Phase 1 don’t allow for data sharing by the lead node.

## Abbreviations

PC: Primary care
OUD: Opioid use disorder
FY: Fiscal year
HER: Electronic health record
AAP: American Academy of Pediatrics
