# Supplementary material for: Receipt of medications for opioid use disorder among youth engaged in primary care: data from 6 health systems
Source: Addict Sci Clin Pract. 2021 Jul 7;16:46. doi: 10.1186/s13722-021-00249-3 (PMC8262000; doi:10.1186/s13722-021-00249-3)
Supplement: Supplementary file 1 — Additional file 1: Table S1. International Classification of Diseases, Ninth and Tenth Revision, Clinical Modification codes used to define Opioid Use Disorder (OUD). [file 13722_2021_249_MOESM1_ESM.docx]

**Additional Table S1. International Classification of Diseases, Ninth and Tenth Revision, Clinical Modification codes used to define Opioid Use Disorder (OUD)**

| Code | Name | ICD9 or ICD10 |
| --- | --- | --- |
| 304 | Opioid Dependence, Unspecified | ICD9 |
| 304.01 | Opioid Dependence, Continuous | ICD9 |
| 304.02 | Opioid Dependence, Episodic | ICD9 |
| 304.03 | Opioid type dependence, in remission | ICD9 |
| 304.7 | Opioid/Other Dependence, Unspecified | ICD9 |
| 304.71 | Opioid/Other Dependence, Continuous | ICD9 |
| 304.72 | Opioid/Other Dependence, Episodic | ICD9 |
| 304.73 | Opioid/other dependence, in remission | ICD9 |
| 305.5 | Opioid Abuse, Unspecified | ICD9 |
| 305.51 | Opioid Abuse, Continuous | ICD9 |
| 305.52 | Opioid Abuse, Episodic | ICD9 |
| 305.53 | Opioid abuse, in remission | ICD9 |
| F11.1x | Opioid abuse related disorders | ICD10 |
| F11.2x | Opioid dependence related disorders, excluding F11.21x | ICD10 |
| F11.21x | Opioid dependence in remission | ICD10 |
