# Supplementary material for: Receipt of medications for opioid use disorder among youth engaged in primary care: data from 6 health systems
Source: Addict Sci Clin Pract. 2021 Jul 7;16:46. doi: 10.1186/s13722-021-00249-3 (PMC8262000; doi:10.1186/s13722-021-00249-3)
Supplement: Supplementary file 2 — Additional file 2: Appendix S1. Prevalence of demographic and clinical characteristics and 95% confidence intervals in youth with opioid use disorder (OUD) with and without documented treatment with medications for OUD stratified by age (N=2131). [file 13722_2021_249_MOESM2_ESM.docx]

|  | 16-17 Years  (n=119) | | 18-21 Years  (n=664) | | 22-25 Years  (n=1348) | | | All Youth  (n=2131) | |
| --- | --- | --- | --- | --- | --- | --- | --- | --- | --- |
|  | **Medication Treatment***  (n=17) | **No Treatment*** (n=102) | **Medication Treatment***  (n=213) | **No Treatment** (n=451) | **Medication Treatment***  (n=522) | **No Treatment** (n=826) | **Medication Treatment***  (n=752) | | **No Treatment*** (n=1379) |
| **------------------------------------------------------------------------------------------- % (95% CI)** ------------------------------------------------------------------------------------ | | | | | | | | | |
| Female | 65 (38, 86) | 54 (44, 64) | 38 (31, 45) | 44 (39, 48) | 37 (33, 42) | 40 (36, 43) | 38 (35, 42) | | 42 (39, 45) |
| Race/ethnicity |  |  |  |  |  |  |  | |  |
| Hispanic | 24 (6.8, 50) | 17 (10, 25) | 7.5 (4.4, 12) | 10 (7.4, 13) | 8.8 (6.5, 12) | 7 (5.4, 9) | 8.8 (6.9, 11) | | 8.7 (7.3, 10) |
| Caucasian | 65 (38, 86) | 69 (59, 77) | 82 (76, 87) | 73 (69, 77) | 79 (75, 82) | 78 (75, 81) | 79 (76, 82) | | 76 (74, 78) |
| Black/African American | 0 (0, 20) | 2.9 (0.6, 8.4) | 1.9 (0.5, 4.7) | 3.3 (1.9, 5.4) | 1.9 (0.9, 3.5) | 4.2 (3, 5.8) | 1.9 (1.0, 3.1) | | 3.8 (2.9, 5) |
| Asian | 5.9 (0.1, 29) | 2 (0.2, 6.9) | 0.9 (0.1, 3.4) | 2.7 (1.4, 4.6) | 1.1 (0.4, 2.5) | 1.7 (0.9, 2.8) | 1.2 (0.5, 2.3) | | 2.0 (1.4, 2.9) |
| NA/ AN | 0 (0, 20) | 0 (0, 3.6) | 0.5 (0.01, 2.6) | 1.1 (0.4, 2.6) | 0.6 (0.1, 1.7) | 0.6 (0.2, 1.4) | 0.5 (0.1, 1.4) | | 0.7 (0.3, 1.3) |
| Hawaiian/Pacific Islander | 0 (0, 20) | 0 (0, 3.6) | 0 (0, 1.7) | 0.2 (0.006, 1.2) | 1.1 (0.4, 2.5) | 0.1 (0.003, 0.7) | 0.8 (0.3, 1.7) | | 0.1 (0.02, 0.5) |
| Multiracial | 5.9 (0.1, 29) | 9.8 (4.8, 17) | 2.8 (1, 6) | 6.2 (4.2, 8.8) | 4.2 (2.7, 6.3) | 3.9 (2.7, 5.4) | 3.9 (2.6, 5.5) | | 5.1 (4, 6.4) |
| Other | 0 (0, 20) | 0 (0, 3.6) | 0.9 (0.1, 3.4) | 0.9 (0.2, 2.3) | 0.8 (0.2, 2) | 1.1 (0.5, 2.1) | 0.8 (0.3, 1.7) | | 0.9 (0.5, 1.6) |
| Unknown | 0 (0, 20) | 0 (0, 3.6) | 3.8 (1.6, 7.3) | 2.7 (1.4, 4.6) | 2.9 (1.6, 4.7) | 3 (2, 4.4) | 3.1 (1.9, 4.6) | | 2.7 (1.9, 3.7) |
| Insurance* |  |  |  |  |  |  |  | |  |
| Medicare | 24 (6.8, 50) | 13 (6, 23) | 8 (4.7, 12) | 12 (8.8, 16) | 12 (9, 15) | 14 (12, 17) | 11 (8.7, 13) | | 13 (11, 16) |
| Commercial | 76 (50, 93) | 85 (74, 92) | 91 (86, 95) | 84 (80, 88) | 83 (80, 86) | 78 (75, 82) | 85 (83, 88) | | 81 (78, 83) |
| State subsidized | 0 (0, 20) | 0 (0, 5.1) | 0 (0, 1.7) | 0 (0, 1.1) | 0 (0, 0.7) | 0.3 (0.04, 1.2) | 0 (0, 0.5) | | 0.2 (0.02, 0.7) |
| Uninsured | 0 (0, 20) | 2.8 (0.3, 9.8) | 0.9 (0.1, 3.4) | 3.6 (1.8, 6.1) | 5 (3.3, 7.3) | 7.1 (5.2, 9.5) | 3.8 (2.5, 5.4) | | 5.6 (4.3, 7.2) |
| Tobacco UD | **12 (1.5, 36)** | **48 (38, 58)** | 59 (52, 65) | 55 (50, 60) | 56 (52, 60) | 51 (48, 55) | 56 (52, 59) | | 52 (50, 55) |
| Alcohol UD | 47 (23, 72) | 45 (35, 55) | 18 (13, 24) | 27 (23, 31) | 22 (18, 26) | 24 (21, 27) | 21 (18, 24) | | 26 (24, 29) |
| Cannabis UD | 71 (44, 90) | 64 (54, 73) | 46 (39, 52) | 38 (34, 43) | 29 (25, 33) | 25 (22, 28) | 34 (31, 38) | | 32 (30, 35) |
| Stimulant UD | 29 (10, 56) | 32 (23, 42) | 35 (28, 42) | 28 (24, 33) | 28 (24, 32) | 25 (22, 29) | 30 (26, 33) | | 27 (25, 29) |
| OD | 5.9 (0.1, 29) | 2 (0.2, 6.9) | 4.7 (2.3, 8.5) | 2.9 (1.5, 4.9) | 2.7 (1.5, 4.5) | 4.4 (3.1, 6) | 3.3 (2.2, 4.9) | | 3.7 (2.8, 4.8) |
| Depression | 76 (50, 93) | 77 (68, 85) | 37 (30, 43) | 47 (42, 51) | **34 (30, 38)** | **44 (41, 48)** | **36 (32, 39)** | | **48 (45, 50)** |
| Anxiety | 65 (38, 86) | 66 (56, 75) | 41 (34, 48) | 49 (44, 53) | 41 (37, 46) | 49 (46, 53) | **42 (38, 45)** | | **50 (48, 53)** |
| SMI | 12 (1.5, 36) | 16 (9.2, 24) | **4.7 (2.3, 8.5)** | **12 (8.7, 15)** | 7.7 (5.5, 10) | 12 (9.5, 14) | **6.9 (5.2, 9.0)** | | **12 (10, 14)** |
| ADD | 18 (3.8, 43) | 34 (25, 44) | 15 (10, 20) | 12 (9.3, 16) | 9.8 (7.4, 13) | 13 (11, 15) | 11 (9.1, 14) | | 14 (12, 16) |
| Eating d/o | 5.9 (0.1, 29) | 2.9 (0.6, 8.4) | 1.4 (0.3, 4.1) | 4 (2.4, 6.2) | 0.4 (0.05, 1.4) | 1.7 (0.9, 2.8) | **0.8 (0.3, 1.7)** | | **2.5 (1.8, 3.5)** |

Appendix S1: Prevalence of demographic and clinical characteristics and 95% confidence intervals in youth with opioid use disorder (OUD) with and without documented treatment with medications for OUD stratified by age (N=2131)
